# Catheter segmentation in X-ray fluoroscopy using synthetic data and transfer learning with light U-nets

**DOI:** 10.1016/j.cmpb.2020.105420

**Published:** 2020-08

**Authors:** Marta Gherardini, Evangelos Mazomenos, Arianna Menciassi, Danail Stoyanov

**Affiliations:** a The BioRobotics Institute, Scuola Superiore Sant’Anna, Pisa, Italy; b Department of Excellence in Robotics & AI, Scuola Superiore SantâĂŹAnna, 56127 Pisa, Italy; c Wellcome/EPSRC Centre for Interventional and Surgical Sciences, University College London, London, U.K; d School of Electronic and Electrical Engineering, University of Leeds, Leeds, U.K

**Keywords:** Catheter segmentation, Deep learning, Fluoroscopy, Transfer learning

## Abstract

*Background and objectives*Automated segmentation and tracking of surgical instruments and catheters under X-ray fluoroscopy hold the potential for enhanced image guidance in catheter-based endovascular procedures. This article presents a novel method for real-time segmentation of catheters and guidewires in 2d X-ray images. We employ Convolutional Neural Networks (CNNs) and propose a transfer learning approach, using synthetic fluoroscopic images, to develop a lightweight version of the U-Net architecture. Our strategy, requiring a small amount of manually annotated data, streamlines the training process and results in a U-Net model, which achieves comparable performance to the state-of-the-art segmentation, with a decreased number of trainable parameters.

*Methods*The proposed transfer learning approach exploits high-fidelity synthetic images generated from real fluroscopic backgrounds. We implement a two-stage process, initial end-to-end training and fine-tuning, to develop two versions of our model, using synthetic and phantom fluoroscopic images independently. A small number of manually annotated *in-vivo* images is employed to fine-tune the deepest 7 layers of the U-Net architecture, producing a network specialized for pixel-wise catheter/guidewire segmentation. The network takes as input a single grayscale image and outputs the segmentation result as a binary mask against the background.

*Results*Evaluation is carried out with images from *in-vivo* fluoroscopic video sequences from six endovascular procedures, with different surgical setups. We validate the effectiveness of developing the U-Net models using synthetic data, in tests where fine-tuning and testing *in-vivo* takes place both by dividing data from all procedures into independent fine-tuning/testing subsets as well as by using different *in-vivo* sequences. Accurate catheter/guidewire segmentation (average Dice coefficient of  ~ 0.55,  ~ 0.26 and  ~ 0.17) is obtained with both U-Net models. Compared to the state-of-the-art CNN models, the proposed U-Net achieves comparable performance ( ± 5% average Dice coefficients) in terms of segmentation accuracy, while yielding a 84% reduction of the testing time. This adds flexibility for real-time operation and makes our network adaptable to increased input resolution.

*Conclusions*This work presents a new approach in the development of CNN models for pixel-wise segmentation of surgical catheters in X-ray fluoroscopy, exploiting synthetic images and transfer learning. Our methodology reduces the need for manually annotating large volumes of data for training. This represents an important advantage, given that manual pixel-wise annotations is a key bottleneck in developing CNN segmentation models. Combined with a simplified U-Net model, our work yields significant advantages compared to current state-of-the-art solutions.

## Highlights

• Fully-automated, real-time catheter and guidewire segmentation in fluoroscopy using CNNs.
• Two-stage training strategy based on transfer learning technique, using synthetic images with predefined labelled segmentation.
• Methods to reduce the need of manual pixel-level labelling to facilitate the development of CNN models for semantic segmentation, especially in the medical field.
• Lightweight CNN model with a decreased number of network parameters which results in more efficient training and faster run times (84% reduction in testing time compared to the state-of-the-art).

## 1 Introduction

The projections of the World Health Organization indicate that cardiovascular diseases (CVDs) will remain, in the near future, the leading cause of death worldwide [1]. Treatment of CVDs often requires surgical intervention and advances in flexible instrumentation and imaging led to the development and application of minimally invasive surgery (MIS) for cardiac and vascular procedures. MIS offers many advantages, including decreased blood loss, reduced post-operative pain and shorter recovery times, and is nowadays the preferred method for CVD interventions [2].

In MIS endovascular procedures, flexible catheters are navigated inside the patient’s vasculature for delivering therapeutic actions under real-time, image-based guidance. Predominately fluoroscopy is used, while echocardiography is also carried out for heamodynamic management. X-ray fluoroscopy involves harmful ionizing radiation and toxic contrast dye injections for vessel visualization and catheter localization. Subsequently, significant research is focused on alternative imaging modalities, without such adverse effects, both pre- and intra-operatively for complementing or even replacing fluoroscopy. Ultrasound (US) imaging presents many advantages, such as the absence of ionizing radiation and richness in soft-tissue information, however it is not suitable for catheter guidance because of acoustic artefacts and limited field of view that hinder catheter visualization in US images. Other imaging modalities with potential to improve endovascular guidance are magnetic resonance imaging (MRI), computed tomography (CT) and 3d rotational X-ray angiography, typically performed pre-operatively. Fusing multiple imaging modalities and developing computer vision techniques to enhance tool visualization can lead to accurate and robust localization of the catheter, with reduced use of fluoroscopy [3], [4], [5], [6], [7], [8], [9], [10]. In many situations this requires registration of 3d pre-operative data to 2d intra-operative fluoroscopy images, of which a key aspect is the ability to accurately localize and segment the catheter in the X-ray fluoroscopic images.

This work presents a novel approach for performing fully-automated, real-time catheter and guidewire segmentation in fluoroscopy. The proposed method is based on a Convolutional Neural Network (CNN) encoder-decoder architecture, termed as U-Net and developed specifically for medical image segmentation tasks [11]. An important requirement in the development of CNNs for pixel-level segmentation is the availability of large numbers of annotated samples to successfully train the network. Manual pixel-level labelling is laborious and time-consuming and methods to reduce this necessity will greatly facilitate the development of CNN models for semantic segmentation. This is particularly important for the surgical imaging domain where annotated medical data are not always available in large volumes. Although CNN architectures have been used before for catheter detection and tracking [12], [13], we differentiate from previous works by proposing a strategy for streamlining the training process that exploits synthetic data and uses only a small amount of annotated images. Specifically, we follow a transfer learning approach, a technique that adds flexibility in training and developing CNNs [14], [15], [16], [17], [18], [19]. In transfer learning, a randomly initialized *base* network is first trained on a base dataset and subsequently the learned features and weights are *transferred* to a *target* network to be refined on a smaller and target dataset. By using synthetic images with predefined labelled segmentation for the initial training phase, our method requires a reduced amount of explicitly annotated samples to be available, only for the refinement stage. In addition, it has been demonstrated that transferring features and fine-tuning them with a different dataset results in network models that generalize better and avoid overfitting than those trained exclusively on the target dataset [20]. The proposed U-Net architecture is based on the model presented in [12]; however, compared to this work, the modifications we apply decrease the total number of network parameters, thus resulting in more efficient training and faster run times. Our transfer learning investigation is carried out on different sets of fluoroscopic images, where synthetic images, requiring minimum manual effort for annotation, and fluoroscopic images from a physical phantom are utilized to train a CNN model that learns to accurately segment endovascular surgical tools in *in-vivo* images.

We perform two experiments, for the initial end-to-end training stage. The first one is carried out with 9000 synthetic fluoroscopic images and the second one with 2000 real fluoroscopic images from catheterization experiments with a silicon aorta phantom in an angiography suite. After achieving convergence, we freeze the network parameters and carry out three different tests by fine-tuning only the deepest layers of the architecture on three different datasets (S1, S2 and S3) selected from 1207 manually labelled, *in-vivo* fluoroscopic images from six different X-ray sequences. For each split (240, 493 and 579 fine-tuning frames in S1, S2 and S3, respectively), the remaining frames were used for testing. High-quality catheter segmentation results were achieved, with an average Dice coefficient value of approximately 0.55, 0.26 and 0.17 for each of the three tests, respectively. To illustrate the need and effectiveness of using synthetic data for the end-to-end training, we present a leave-one-out (LOO) 6-fold cross validation experiment with training and testing carried out using only the *in-vivo* sequences. Due to the limited number of training data, the resulting network is not able to effectively learn the catheter segmentation task (Dice coefficient  ~ 0.1). Moreover, we compare our modified U-Net network, with the one presented in [12], after reducing the number of layers to adapt it to our input images, and demonstrate that the two models achieve comparable segmentation performance. Our U-Net model has a smaller number of network parameters, offering several benefits from a practical implementation perspective [21]. This also results in faster execution times, with the proposed model requiring an average of 71 ms to segment a single image, instead of 451 ms needed by the model in [12] when tested in our setup. This is equivalent to an 84% reduction of the testing time.

In summary, the main contributions introduced in this work are the following:

1. We demonstrate the effectiveness of transfer learning in developing a CNN model for segmenting surgical catheters in fluoroscopy images. In the absence of large annotated real medical datasets, the end-to-end training of a lightweight U-Net model is streamlined by using high-fidelity synthetic data with available ground truth. A small number of explicitly annotated images is employed to fine-tune the deepest layers of the network, providing a model that is capable to accurately perform catheter segmentation.
2. We present an optimized U-Net CNN architecture, trainable end-to-end to perform fully-automated, real-time catheter segmentation in 2d fluoroscopy images. The proposed model achieves comparable performance to the state-of-the-art segmentation while the reduced number of trainable network parameters results in faster execution times.

## 2 Background

Automated segmentation of electrodes and catheters in electrophysiology (EP) procedures is a very active topic in the medical image analysis literature. EP electrodes are more visible under X-ray imaging and are generally exploited as a starting point for catheter segmentation [5], [13], [22]. In [22], after a pre-processing step, sparse coding is employed to initially detect candidate catheter tips and subsequently perform detection and tracking of the catheters; Wu et al. [5] presented a method in which electrodes are first detected using SURF (speeded up robust features) and a Kalman filter is then employed to extend the detection to the entire catheter. Another approach was proposed by Baur et al. [13], in which a fully convolutional neural network is used to detect catheters tips and electrodes; however, full catheter shape segmentation is not implemented. Due to the nature of the fluoroscopy images, catheter segmentation is a challenging task, especially in situations where clearly distinguishable features are not present. A number of methods use Hessian filters followed by spline fitting [23], [24], [25], [26], [27], [28], but none of these is fully-automated, thus requiring manual initiliazation. Wagner et al. proposed a new approach in which the guidewire segmentation is performed through a fully-automated algorithm that exploits a ridge detection filter, noise reduction for curvilinear structures as well as an a-priori probability map, but the complexity of this method makes it unsuitable for real-time applications [29]. Ma et al. [30] proposed to use a vessel enhancement filter for centerline extraction followed by object classification, to detect different types of catheters.

In recent years CNNs revolutionized many computer vision applications achieving state-of-the-art results in tasks such as classification, segmentation and detection. The core idea is to architect models with many interconnected hidden layers, capable of learning multiple levels of abstract representations by being exposed to labelled examples. Development of CNNs involves a training phase during which model parameters are iteratively optimized using large amounts of labelled data. It has been demonstrated that end-to-end training for pixel-to-pixel semantic segmentation tasks presents many benefits [31], although a large amount of training data is typically needed. Following this principle, Ronneberger et al. introduced a new CNN architecture, named U-Net, that is trainable in an end-to-end manner and specifically designed to perform medical image segmentation tasks [11]. In the past years, the U-Net model has been extensively applied in this field [32], [33]. Oktay et al. [32] integrated an attention gate model for medical imaging into a U-Net architecture and applied the resulting model to a large CT abdominal dataset for multi-class image segmentation; Matuszewski et al. [33] recently proposed a reduced-sized U-Net architecture for performing virus recognition in electron microscopy images.

Several modifications to the original network have been applied to improve network convergence (batch normalization [34] and residual learning [35]) and performance (max-pooling replaced by strided convolution [36]). An adapted U-Net model including the aforementioned improvements was proposed by Ambrosini et al. [12], for fully automated, real-time catheter segmentation in 2d X-ray fluoroscopic sequences. The network is applied to video recordings using as inputs the current frame and the three previous ones, and it outputs a segmentation map of the catheter/guidewire in the current image. From the catheter segmentation result, a centerline model of the catheter is then constructed. In comparison to previous studies, this method doesn’t continuously track the catheter, but incorporates temporal information as the network’s input is a sequence of four frames. The model is developed on a training dataset consisting of 182 sequences of four consecutive frames, extracted from *in-vivo* fluoroscopy videos, acquired during 23 liver catheterization procedures (728 frames in total); it is then validated on *in-vivo* images consisting of 55 similar sequences (i.e., 220 frames) from 5 different procedures of the same kind. To provide the ground truth the catheter was manually segmented in the four consecutive images by selecting points and fitting a spline function. Data augmentation was applied to increase the number of training samples.

As mentioned previously, manual pixel-to-pixel annotation is time-consuming and laborious. Considering the amount of labelled images required for end-to-end training, this becomes an even more difficult task. Strategies to reduce the amount of manual annotation carry significant potential for a more efficient, time-saving, end-to-end training of CNN models. In this work we propose the use of synthetic fluroscopic images for training a CNN model to perform catheter segmentation. With this approach we avoid the need for excessive manual annotations since the ground truth segmentation information of both the catheter and background is readily available. Similar strategies have been successfully applied in medical image processing for improving image classification in comparison to classic data augmentation approach [37], as well as in other application fields such as robotics control [38].

The remainder of the paper is structured as follows. In Section 3 we describe the proposed network architecture, detail the generation of the synthetic dataset and the approach we followed for training and fine-tuning the CNN model. Section 4 presents the experimentation we carried out alongside a discussion of the obtained results that justify our strategy and methods. Finally, Section 5 draws conclusions and discusses opportunities for future research.

## 3 Materials and methods

### 3.1 Proposed CNN model

**Fig. 1:**
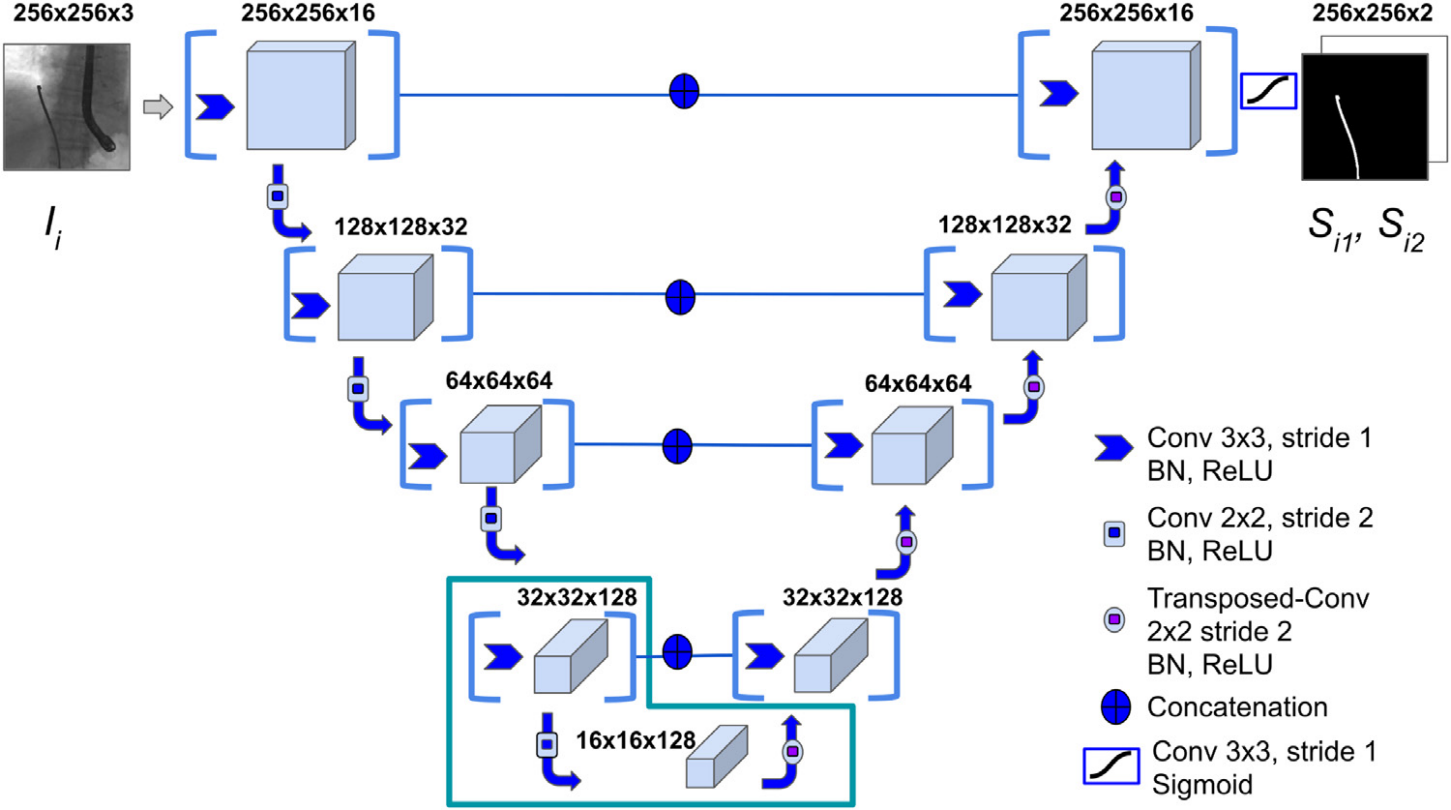
The proposed lightweight U-Net architecture. A single grayscale Image *I_i_* is fed into the model, that outputs the predictions *S*_*i*1_ and *S*_*i*2_. Each layer is composed by a convolutional-block with a 3x3 kernel. The L-shape at the bottom of the architecture delimits the layers that undergo fine-tuning.

Starting from the basic U-Net architecture [11], we develop an adapted version, shown in Figure 1, tailored for catheter segmentation in fluoroscopy images. We structure our model lowering the number of convolution operations per layer from two to one. This simplifies the architecture reducing the total number of learnable parameters. Convolution layers are followed by Batch Normalization and a Rectified Linear Unit (ReLU) is used as activation. In the last convolution layer, a sigmoid activation function is applied to provide a per-pixel classification output in the [0, 1] range. In addition, by lowering the resolution of input images (256x256 px instead of 1024x1024 px), the total number of layers in our design is reduced as well. All in all, the proposed model has 55 layers in total, compared to the 110 layers of the architecture in [12]. Differently from [12], we choose a single grayscale image *I_i_* as the input to our proposed network. The output is given as two complementary full-scale (256x256 px) classification masks (*S*_*i*1_ - catheter, *S*_*i*2_ - background), from which the accuracy of the output is evaluated using the Dice coefficient. In *S*_*i*1_ and *S*_*i*2_ the k-th pixel *s_k_* assumes complementary probability values between 0 and 1. In *S*_*i*1_ (and complementary in *S*_*i*2_), each pixel is ultimately classified as either catheter (1) or background (0) using thresholding as follows:

(1) $$s_{k}=\left\{ \begin{array}{cc} 1, & \mathrm{if}\;\;s_{k}^{p}\;\;\mathrm{is}\;>0.01\\ 0, & \mathrm{if}\;\;s_{k}^{p}\;\;\mathrm{is}\;\leq 0.01 \end{array}\right.$$

### 3.2 Datasets

**Fig. 2:**
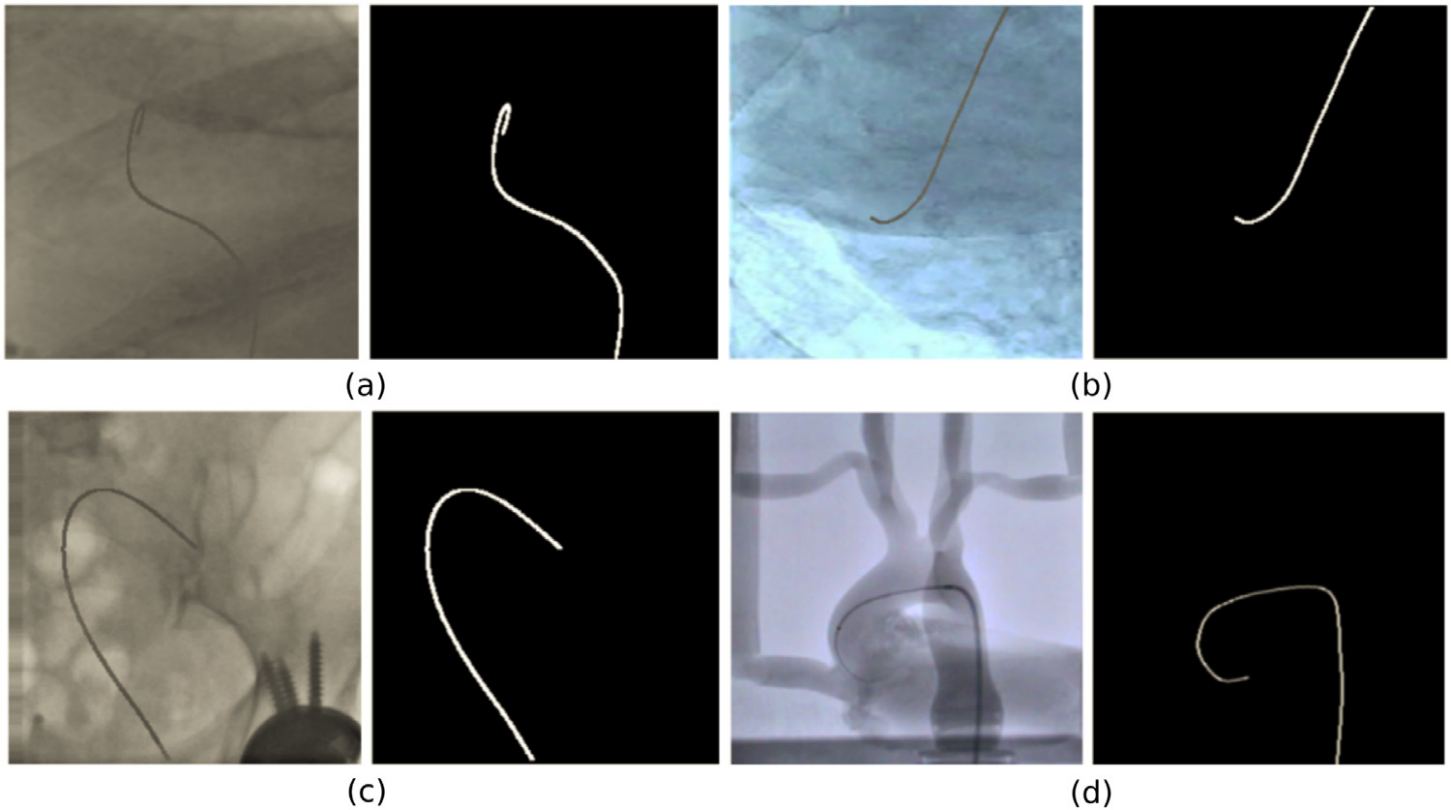
Representative examples of synthetic data (a, b, c) and fluoroscopic phantom image (d) with corresponding catheter ground truth masks.

Three different datasets, listed in Table 1, were used for developing and validating our network. *Dataset-1* is composed of 9000 synthetic fluoroscopic images, generated following the procedure described in the next paragraph. *Dataset-2* consists of 2000 images extracted from four fluoroscopy videos, recorded in an angiography suite, from catheterization experiments carried out on a silicon aorta phantom. Example images from both datasets are shown in Figure 2. *Dataset-3* contains images extracted from six fluoroscopy videos obtained during *in-vivo* endovascular operations. Specifically, 836 frames were extracted from four Transcatheter Aortic Valve Implantation (TAVI) procedures (datasets T1-T4) and 371 from two diagnostic catheterization sequences (datasets T5-T6). Segmentation annotations of the catheter and background were automatically generated for the synthetic images of *Dataset-1*, while for *Dataset-2* and datasets T2-T6 of *Dataset-3* a semi-automated tracking method presented by Chang et al. [24] was employed to obtain the annotations as the 2d coordinates of the catheter restricted to a manually selected region of interest (ROI). Our annotation process is similar to the one in [12], since the algorithm in [24] also employs a b-spline tube model as a prior for the catheter shape in order to restrict the search space and deal with potential missing measurements. This is combined with a probabilistic framework that estimates the pixel-wise posteriors between the foreground (catheter) and background delimited by the b-spline tube contour. The method was tested on both phantom and clinical TAVI sequences, achieving an average missing-rate lower than 4% of the pixels over the entire tracked catheter length. Given the accuracy and robustness of the algorithm, we consider the obtained results as the ground truth catheter segmentation while potential errors will have negligible influence on the development and evaluation of our U-Net model. Finally, for dataset T1 the shape of the entire catheter was manually annotated using crowdsourcing. All annotations consist of full-scale (256x256 px) binary masks where background pixels have a “0” value, while a value equal to “1” denotes the catheter pixels.

**Table 1:** The three datasets used in this work.

Dataset	Data Type	Number of Images
Dataset-1	Synthetic	9000
Dataset-2	Phantom	2000
Dataset-3	*In-vivo* procedures	1207

**Fig. 3:**
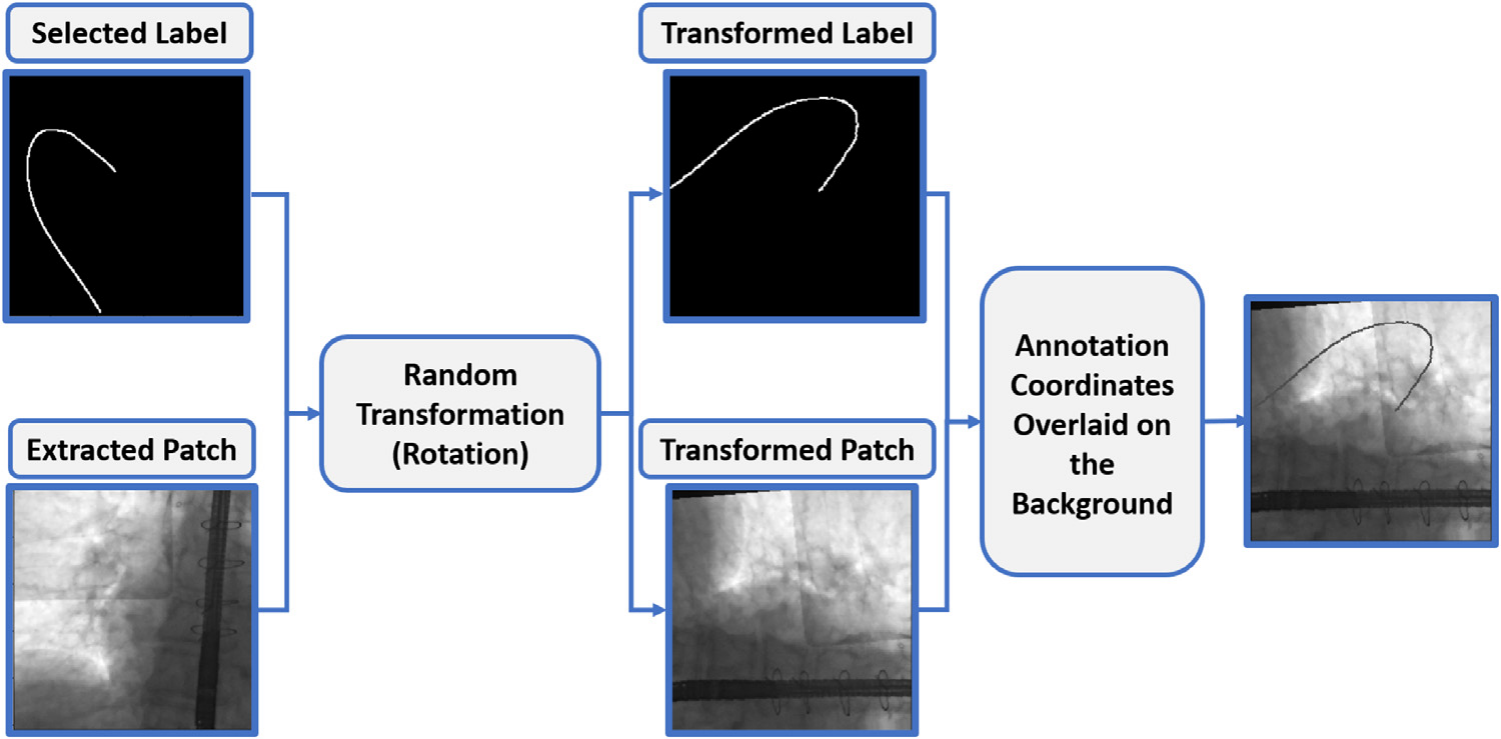
The process for synthetic data generation. A background patch and a binary mask from *Dataset-3* first undergo a random transformation (in this case, rotation). The transformed catheter coordinates are then mapped to the new background to generate the synthetic image.

To generate the synthetic images for *Dataset-1*, 32 patches (image sub-areas) containing no catheter part were extracted from different frames of *Dataset-3*. These patches were selected as representative backgrounds of X-ray images from endovascular interventions, in which the relevant anatomical structures (e.g. bones, organs) and sometimes the US probe used for echocardiography, are visible. Since our strategy is to investigate the applicability of synthetic data for transfer learning, it is important that images in *Dataset-1* resemble, as much as possible, the real fluoroscopic images used in the fine-tuning phase. Indeed, it has been demonstrated that transferability of features decreases as the dissimilarity between the base task (training data) and the target task (fine-tuning data) increases [20]. Extracted background patches were resized to the network’s input resolution (256 x 256 px). A small number of ground truth catheter masks from *Dataset-2* and *Dataset-3* ( ~ 20% the available annotations from each dataset) were selected. The background patch and 2d catheter coordinates were randomly paired and each pair underwent a random transformation, among random rotation (rotation, [0, 90^∘^]), scaling (zoom, [-0.6, 1.4]), horizontal/vertical shift (width shift / height shift, [-0.2, 0.2]) and horizontal/vertical flip. The transformed ground truth coordinates were subsequently used to overlay the catheter shape on the background. The intensity of each catheter pixel was set to the average value of the background intensity plus a random value in the [-50, 50] range to simulate the discontinuous appearance of catheters in fluoroscopy. With this method we generated 9000 high-fidelity synthetic fluoroscopic images for which ground truth segmentation was directly available. (Figure 3). In our synthetic images the catheter was inserted in different positions and with different orientations, as well as having varying pixel values compared to the background. This approach provided the necessary variability in catheter shape and appearance, facilitating the training of the U-Net network.

### 3.3 Training strategy

We train our CNN model using two different cost functions and compare the obtained segmentation accuracy. In the first case, the Dice coefficient is used as both the accuracy metric and loss function. Objective functions based on the Dice coefficient were first introduced by Milletari et al. [36] and its value ranges between [0,1], representing the overlap between the output mask of the network and the annotated segmentation. Using the Dice coefficient, the loss function is defined as:

(2) $$L_{dice}\left(S_{i},S_{i}^{p}\right)=-\frac{2\sum_{k}S_{ik}S_{ik}^{p}+c}{\sum_{k}S_{ik}+\sum_{k}S_{ik}^{p}+c}$$

where, $S_{ik}^{p}$ is the network prediction for the *k_th_* pixel, with values in the range [0,1], *S_ik_* is the corresponding binary label and *c* is a constant smoothing term to avoid division by zero. In the second case, the accuracy is again evaluated with the Dice coefficient, but the categorical-cross-entropy is used as the loss function. Considering that each pixel belongs to exactly one class (i.e. catheter or background), the second cost function is given as:

(3) $$L_{c-e}\left(C,S_{ik}\right)=-\frac{1}{N}\sum_{k}\log p_{k}\left[S_{ik}\in C_{S_{ik}}\right]$$

where, *N* is the total number of pixels in the image and *p_k_* is the probability of the *k_th_* pixel belonging to the $C_{S_{ik}}$ class. Since the number of catheter pixels is significantly smaller compared to the background (i.e., the catheter class is under-represented), both loss functions were assigned a 1/10 ratio between the background and the foreground (catheter) pixel weights during training. The Adam algorithm (learning rate lr=0.001, exponential decay rate for the first moment estimates $\beta_{1}=0.9$ and exponential decay rate for the second-moment estimates $\beta_{2}=0.999$) was used for network weights optimization. For the initial end-to-end training, a batch size of 30 frames was used, and the training process was ended after 12 epochs.

Training images were randomly shuffled and normalized using:

(4) $$p_{kh}^{N}=\frac{p_{kh}-m_{h}}{s_{h}}$$

where *p_kh_* is the *k_th_* pixel of the *h_th_* channel of the input image, *m_h_* is the average pixel intensity and *s_h_* is the intensity standard deviation, both computed for each channel on the entire training set. We train our U-Net model in an end-to-end manner for performing catheter segmentation on a single fluoroscopy image. With this approach and by employing transfer learning using synthetic data, we provide a lighter CNN for catheter segmentation and a more efficient training strategy that effectively differentiates our work from the one in [12], where successive frames from the same fluoroscopy video sequence were used to train the U-Net model.

## 4 Experiments and results

The CNN model was built using the Keras and Tensorflow frameworks in Python and experimentation was carried out in a single NVidia GeForce GTX 1080 GPU. Our investigation focuses on developing an efficient CNN model for real-time catheter segmentation and validating the core contributions of our work, listed in Section 1. Focusing on four directions we performed the following experiments:

1. For determining the optimum training setup and demonstrating the potential of synthetic data, two experiments are discussed in Section 4.1. In *Experiment-1* the network was trained on synthetic data (*Dataset-1*), while in *Experiment-2*, training was performed with the phantom fluoroscopy images (*Dataset-2*). In both situations fine-tuning takes place with *in-vivo* images (*Dataset-3*).
2. For validating the efficiency of the transfer learning and fine-tuning techniques, accuracy results on a test dataset comprising of *in-vivo* fluoroscopy images (*Dataset-3*) and a comparison with and without fine-tuning, for both experiments, are presented in Section 4.2.
3. For illustrating the usefulness of the synthetic data for the initial end-to-end training, we provide a 6-fold cross validation LOO experiment using only the 6 *in-vivo* sequences and compare the results to our transfer learning approach.
4. Finally, in Section 4.3, we show that the proposed lightweight CNN and the more complex state-of-the-art model in [12] achieve comparable performance.

The usefulness of employing a large synthetic dataset for the initial end-to-end training is validated in a LOO validation experiment where only *Dataset-3* is used for training and testing the U-Net model, without fine-tuning. With the available six *in-vivo* sequences, we follow a LOO strategy for training the network on five of them and testing on the remaining one. The number of images used for training ranged between 921 and 1064, and for testing between 143 and 286. The training set-up was the one discussed in Section 3.3. The average Dice coefficient was  ~ 0.1 (range: $1\times 10^{-4}$ - 0.29), listed in Table 4, indicating that due to the limited training set in each fold, the U-Net model is not able to learn the necessary features for the catheter segmentation task. To overcome this, we perform the initial end-to-end training using the large number of synthetic images and, following a fine-tuning step on a small number of *in-vivo* images, develop a model that can effectively segment the catheter.

### 4.1 Training and fine-tuning

To identify the optimum number of layers for fine-tuning, we perform end-to-end training using the synthetic dataset (*Dataset-1*), as discussed in Section 3.3 and with the U-Net model having converged to a set of weights, test three fine-tuning configurations. We use *Dataset-2* of phantom fluroscopic images for this investigation and compare Dice coefficient results, shown in Table 2, from three experiments where 19-layers, 13-layers and 7-layers were symmetrically chosen for fine-tuning with respect to the encoding and decoding part of the network. The deepest layers of the encoder/decoder U-Net model were chosen because they encode characteristics specifically related to the presented data and ultimately learn the most distinctive features for the catheter segmentation task. The Adam algorithm (learning rate lr=0.001, exponential decay rate for the first moment estimates $\beta_{1}=0.9$ and exponential decay rate for the second-moment estimates $\beta_{2}=0.999$) was again used to optimize the weights of the selected layers. A batch size of 10 samples was used and the final weights were derived after 50 epochs of training. Since the three configurations resulted in comparable accuracy, the one with the smaller number of trainable layers (7) was selected. To avoid overfitting, the weights of the layers not selected for fine-tuning were left unchanged.

**Table 2:** Training accuracy for different fine-tuning configurations. The network is first trained on *Dataset-1* and *Dataset-2* is used to fine-tune the selected layers. The 7-layer configuration with the minimum number of trainable parameters is selected.

Configuration	Dice Coefficient
19-layers	0.875
13-layers	0.873
7-layers	0.863

Having identified the optimum set of layers for finetuning (layers 22th to 29th), we develop two U-Net models (*Experiment-1* - training on *Dataset-1, Experiment-2* - training on *Dataset-2*) and evaluate the segmentation performance of the network by dividing *Dataset-3*, containing fluoroscopic sequences from endovascular surgical procedures, into three different splits of fine-tuning/testing sets. In split S1, fine-tuning is performed on 240 randomly selected images, obtained from each subset (T1-T6) of *Dataset-3*, as listed in Table 3. In splits S2 and S3, subsets T1, T4 and T6 (714 images) and subsets T1, T2 and T5 (579 images) are used for fine-tuning respectively. In all splits the remaining images, 967(S1), 493(S2), 628(S3), are used for testing.

**Table 3:** Number of frames respectively used in the fine-tuning (S1) and in the testing phase for each group of data.

Dataset	Fine-Tuning Data	Testing Data
T1	55	231
T2	30	120
T3	40	160
T4	40	160
T5	30	113
T6	45	183

**Table 4:** Segmentation accuracy on the leave-one-out (LOO) experiment and on the network fine-tuned on splits S2 and S3, for both *Experiment-1* and *Experiment-2*. Each row reports the Dice coefficient separately for each testing dataset, as well as the average Dice for each test.

Test	T1	T2	T3	T4	T5	T6	Avg.
**LOO**	0.03	0.29	0.20	$1\times 10^{-4}$	$1\times 10^{-4}$	0.10	0.10
**S1 - Exp. 1**	-	0.31	0.31	-	0.16	-	0.26
**S2 - Exp. 1**	-	-	0.36	0.02	-	0.11	0.15
**S1 - Exp. 2**	-	0.28	0.33	-	0.15	-	0.25
**S2 - Exp. 2**	-	-	0.36	0.07	-	0.13	0.19

**Fig. 4:**
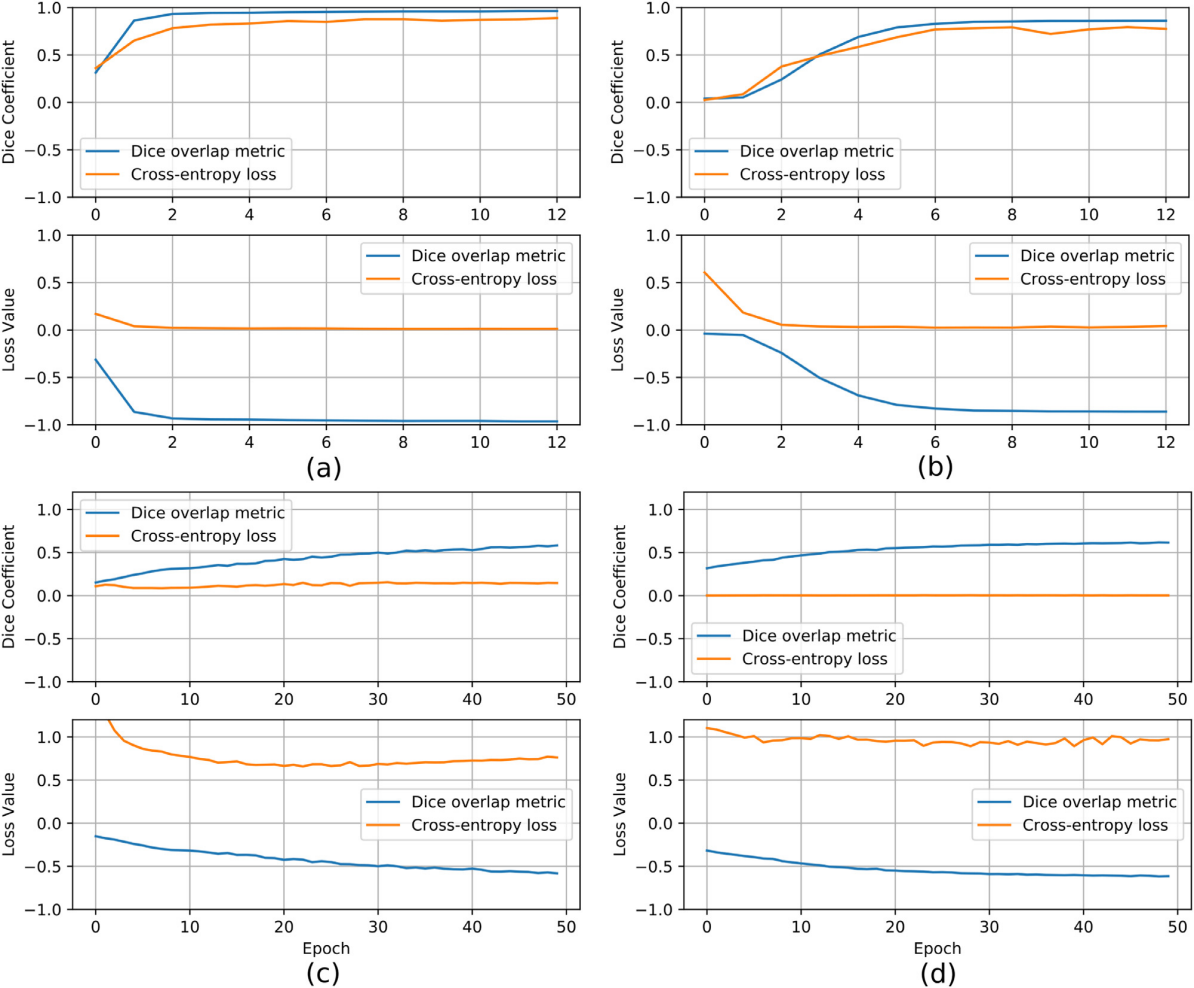
Loss and accuracy values, using Dice coefficient (blue) and categorical-cross-entropy (orange) losses, against the number of epochs for training (a,b) and fine-tuning on S1 (c,d) on synthetic data (a, c - *Experiment-1*) and phantom data (b, d - *Experiment-2*).

As discussed in Section 3.3 we evaluated two cost functions in the development of our U-Net model. Training loss and accuracy values before and after fine-tuning using the S1 split for both *Experiment-1* and *Experiment-2* are illustrated in Fig. 4. It is evident that the two cost functions produce different training outcomes. Indeed, although the end-to-end training proved effective using both cost functions, better performance is achieved with the Dice overlap metric. Specifically, 0.97 (0.85) accuracy on the validation set was derived in *Experiment-1* (*Experiment-2*) when the Dice overlap metric was used, against the 0.87 (0.80) obtained with the cross-entropy loss. More importantly, in the fine-tuning phase, the cross-entropy loss function showed a dramatic drop in performance, with an accuracy on the validation set of about 0.14 (0.02) in *Experiment-1* (*Experiment-2*), as opposed to the 0.58 (0.61) achieved with the Dice overlap metric. These results highlight the importance of selecting the proper cost function in order to develop a model able to generalize in unseen data. For this reason, only the CNN model trained with the Dice overlap metric is used for further experimentation (evaluation on the testing dataset, comparison with the state-of-the-art).

### 4.2 Lightweight U-Net accuracy results

**Fig. 5:**
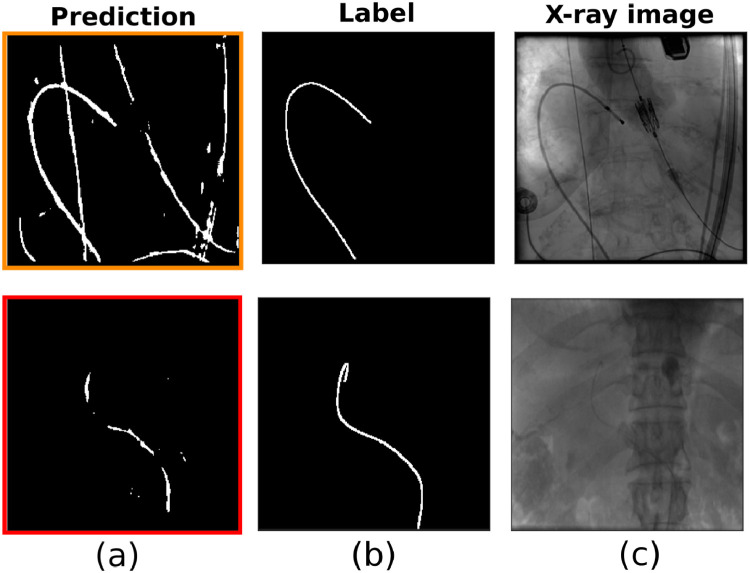
Segmentation results on two representative frames from T2 (upper panel) and T5 (lower panel), with the network fine-tuned on S1 (*Experiment-1)*. The figure shows: (a) the network prediction, (b) the ground truth mask, (c) the corresponding X-ray image.

**Fig. 6:**
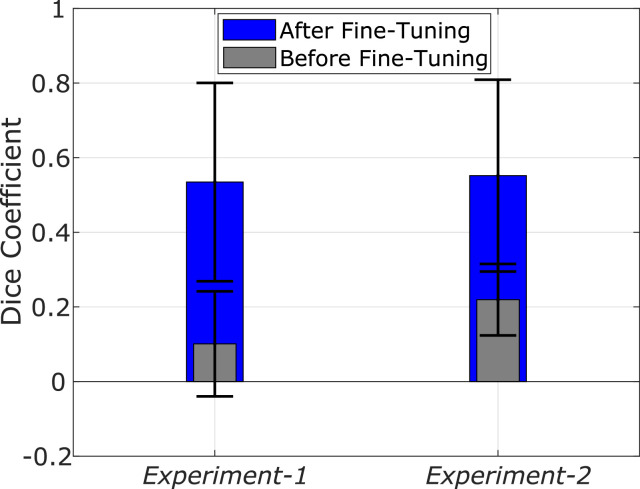
Testing accuracy (average Dice coefficient) before (grey) and after (blue) fine-tuning on S1 for *Experiment-1* and *Experiment-2*. Accuracy increased by  ~ 0.81 and  ~ 0.60, respectively in *Experiment-1* and *Experiment-2*, after fine-tuning.

Segmentation accuracy results for both *Experiment-1* and *Experiment-2* of the lightweight U-Net model are reported in Table 5 for S1, and Table 4 for S2 and S3. Fig. 5 also shows the network prediction for two representative frames from T2 and T5, respectively, when the network is fine-tuned on S1 (*Experiment-1*). In both *Experiment-1 and Experiment-2*, the segmentation is more accurate in split S1 (average Dice coefficient of 0.55) than in S2 and S3 (average Dice coefficient 0.25 and 0.17, respectively). In all fine-tuning/testing configurations the accuracy values obtained in the testing phase is comparable for the two experiments. We therefore argue that a similar training outcome is produced with both the synthetic and the fluoroscopic phantom images. Fig. 6 illustrates the average Dice coefficient computed on the six test subsets (T1-T6) of *Dataset-3*, for both experiments, before and after fine-tuning with the S1 split. The observed net improvement in the segmentation accuracy ( ~ 81% and  ~ 60% increase in *Experiment-1* and *Experiment-2*, respectively) highlights the efficiency of combining transfer learning, using synthetic images, with a fine-tuning step in specializing a network on the target testing set. Example segmentation results on images from *Dataset-3* and the U-Net model developed with the S1 split, are illustrated in Fig. 8. From these we argue that the network learns to discriminate the catheter from other tools (such as the ultrasound probes or surgical screws) as well as from anatomical osseous structures (e.g. ribs, spinal cord), with only a small number of pixels (see Table 5) misclassified as false positives (FPs). For dataset T1, in which all catheters are manually annotated, the network is also capable of segmenting multiple catheters present in the same image (first row in Fig. 8). Interestingly, in dataset T2, the network segments parts of catheters which are outside of the labelled ROI, indicating the model’s ability to generalize. From Table 5 we observe that segmentation performance decreases on T5 and T6 for both experiments. Fig. 8 shows examples from T5 and T6 where the catheter shape is not completely segmented. This is attributed to the low contrast of sequences T5 and T6, making the catheter segmentation challenging, and also to the presence of black borders in T6 that further affects the segmentation result. Despite this, only a small number of FPs exists in T6 (see Table 5).

**Table 5:** Segmentation results for the network fine-tuned on S1. Dice coefficient and percentage of misclassified pixels in terms of False Positives (FPs) for *Experiment 1* (CNN trained on synthetic data) and *Experiment 2* (CNN trained on phantom data), after fine-tuning on S1.

Dataset	Dice Exp.1	Dice Exp.2	FPs (%) Exp.1	FPs (%) Exp.2
T1	0.58	0.57	0.98	1.18
T2	0.78	0.76	2.35	2.85
T3	0.72	0.77	1.08	1.14
T4	0.71	0.73	1.82	2.55
T5	0.29	0.29	1.48	1.97
T6	0.13	0.18	1.33	2.10

### 4.3 Comparison with state-of-the-art model

**Fig. 7:**
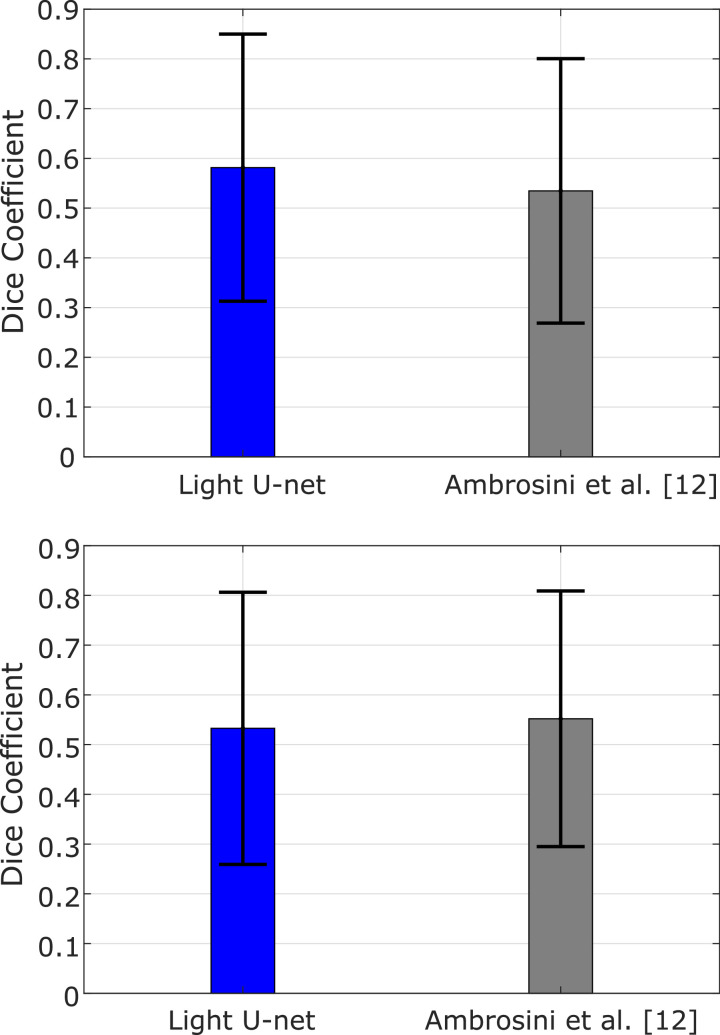
Testing accuracy (Dice coefficient) between the proposed lightweight U-Net architecture and the one in [12] for *Experiment-1* (top) and *Experiment-2* (bottom).

In order to further demonstrate the effectiveness of our approach, we compare the proposed U-Net architecture with the state-of-the-art model for catheter segmentation. Using our datasets, *Experiment-1* and *Experiment-2* are also performed with the model introduced in [12], adapted to our input resolution. Training was followed by refinement of the deepest 7 layers on the S1 split (240 images,  ~ 25% of *Dataset-3*), while the remaining samples (967 images,  ~ 75%) were used for testing. Fig. 7 shows the average Dice coefficient computed on the testing data from T1-T6, for both architectures and both experiments.

**Fig. 8:**
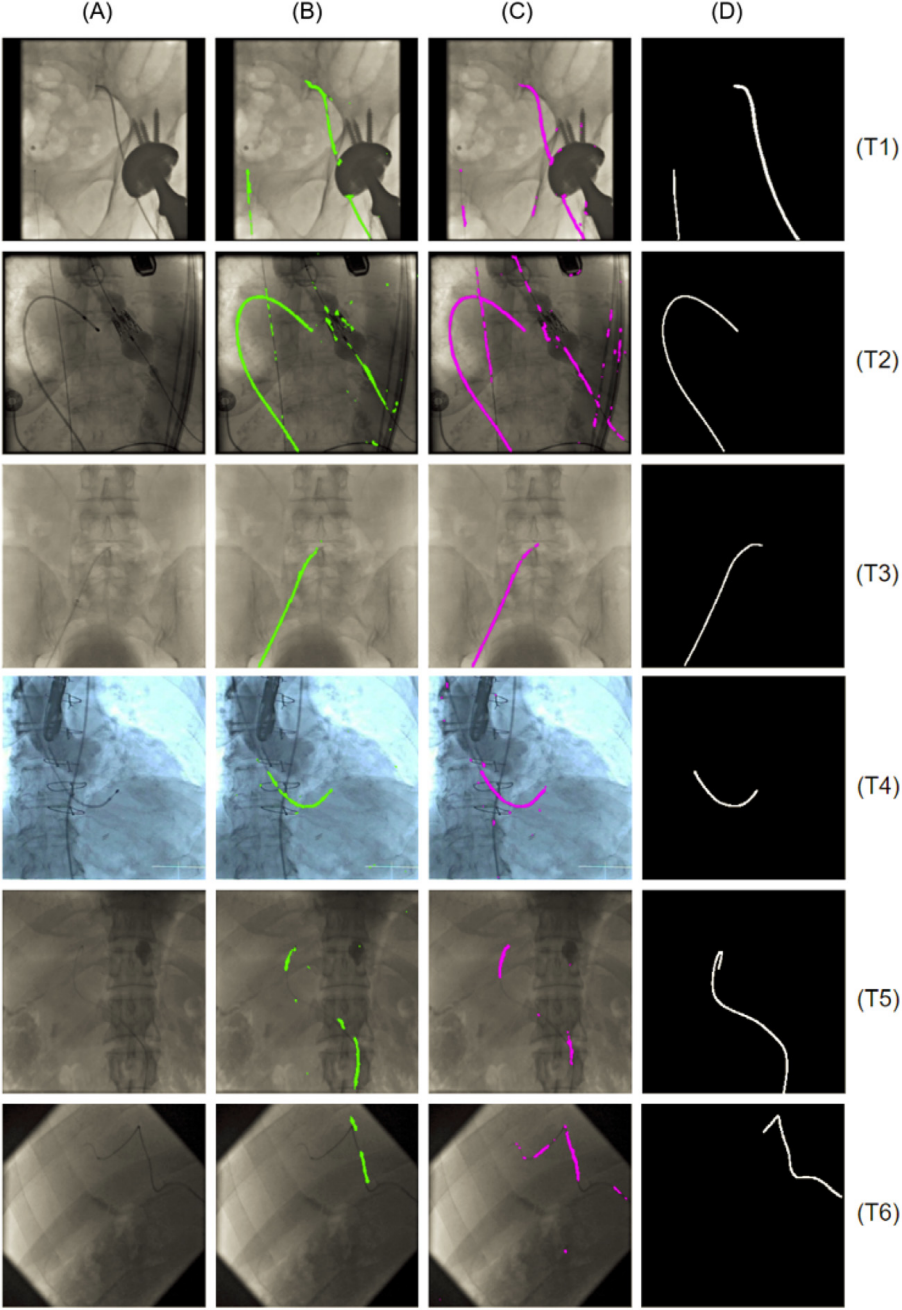
Segmentation results on example images from all test subsets (T1-T6) after fine-tuning on the S1 split: (A) Input grayscale image; (B) Output of *Experiment-1* (CNN trained on synthetic data); (C) Output of *Experiment-2* (CNN trained on phantom data); (D) Ground truth mask: Model’s ability to generalize indicated by accurate segmentation in presence of additional tools (T2), the ultrasound probe (T4), surgical screws (T1) and osseous structures (T3, T5). In T1, the network successfully segments multiple catheters present in the same image and in T2, the network segments catheter parts outside of the labelled ROI.

In *Experiment-1* our lightweight U-Net architecture showed an average Dice coefficient of 0.58, which is higher than the 0.53 obtained with the state-of-the-art model. In *Experiment-2*, a 0.53 average Dice coefficient was derived from the proposed model, compared to the 0.55 of the state-of-the-art. On average, there is a difference in terms of accuracy of 5% for *Experiment-1* and 4% for *Experiment-2*.

Overall, the two models give comparable results and both have high variability in the accuracy on different datasets (see error bars in Fig. 7). We therefore argue that it is possible to reduce the number of trainable parameters of the U-Net model while preserving the same accuracy level, with the additional advantage of a simplified CNN architecture that leads to faster execution times. When tested in our setup, the testing time needed by our simplified U-Net model (reported in Table 6) is reduced by 84% compared to that required by the model presented in [12]. This makes our proposed architecture ideal for real-time operation and more scalable to possible future modifications of the input (e.g. increased image resolution).

**Table 6:** Number of trainable parameters and average testing time for the proposed lightweight U-Net and the one in [12].

CNN architecture	#Parameters	Average Time
Ambrosini et al. [12]	14,133,154	451 ± 12 ms
Light U-Net	687,634	71 ± 2 ms

Similarly to the evaluation performed in [12], we also test the model from *Experiment-1* on fluroscopic video sequences from *in-vivo* TAVI operations (subset T1). We demonstrate that our proposed simplified U-Net architecture, trained on a collection of synthetic images, is able to accurately segment the catheter in sequential frames from endovascular procedures (please see supplementary material for visualizing the original TAVI video and corresponding output segmentation results).

## 5 Discussion and conclusion

The proposed lightweight U-Net architecture trained end-to-end with 9000 synthetic fluoroscopic images (*Experiment-1*), and subsequently fine-tuned on a small number of manually annotated data achieves accurate segmentation performance in most experimental splits. The superior performance (average Dice coefficient > 0.50) is achieved in S1 when a small number (25%) from each available fluoroscopic sequence is used for fine-tuning and the remaining (75%) for testing. Given the reduced size of the *in-vivo* dataset and the fact that fine-tuning may not be very efficient (i.e. transferability of features) if the fine-tuning and testing data differ significantly, this experimental split (S1) provides a benchmark of performance for our network and allows a direct comparison with the state-of-the-art network in [12], developed with images from the same procedure.

Varying segmentation performance is observed in splits S2 and S3 for the different sequences (T1-T6). Good segmentation is obtained for T2 and T3 (Dice coefficient > 0.30), whereas in the remaining cases and particularly in T4 (Dice coefficient < 0.1) the accuracy diminishes. We attribute this to the fact that data in T1-T6 were not only acquired from different types of endovascular procedures, but also from different medical setups (different surgical tools and imaging devices), thus the fluoroscopic images have different characteristics. Particularly evident is the case in T4 where the image contrast is characteristically different than that of the other sequences.

The benefit of the proposed transfer-learning approach using the synthetic dataset is confirmed by the inability of the model to learn the segmentation task as illustrated with the cross validation LOO experiment, where training is performed using only the *in-vivo* fluoroscopic images (*Dataset-3*). The need for large datasets for end-to-end training of deep networks is confirmed, and with our proposed approach we provide an effective strategy for overcoming limitations on data availability.

As discussed in Section 3.2, synthetic data were generated from annotations and background patches extracted from *Dataset-3*. In a small number of situations, the semi-automated tracking method of [24], which employs a ROI for performing catheter tracking, is restricted to a portion of the catheter. The T4 result of Fig. 8 presents one such example, where the ground truth mask annotates a significant, but not complete part of the entire catheter. Nevertheless, in the majority of the available annotations, the obtained masks include the entire visible part of the catheter in the fluoroscopic image. We therefore consider the ground truth masks we obtained to be accurate and suitable for our investigation. This article presents a novel approach for performing pixel-wise segmentation of surgical catheters in 2d X-ray fluoroscopy images. We demonstrate the applicability of using synthetic data and a streamlined training strategy for deep CNN networks intended for performing focused tasks. With the proposed transfer learning approach the amount of manually annotated data for training CNNs can be significantly reduced (only 240, 493 and 579 images were used for fine-tuning). We also show that our simplified U-Net architecture trained on randomly-presented samples achieves comparable accuracy to the state-of-the-art CNN models for catheter segmentation, with an average Dice coefficient difference within 4%-5%, and can adequately segment the catheter on fluoroscopic videos from real endovascular procedures (results provided in the supplementary material).

Potential areas of future work include further investigation into the use of synthetic data for training and the application of more complex CNN architectures. In addition, post-processing techniques applied to the output segmentation mask, like extracting the catheter’s centerline, can be exploited to improve the segmentation outcome and robustness of the method, particularly in challenging situations with low-contrast fluoroscopic imaging.

## Declaration of Competing Interest

All authors declare no conflict of interest.

## Appendix A Supplementary materials

Supplementary Data S1 Supplementary Raw Research Data. This is open data under the CC BY license http://creativecommons.org/licenses/by/4.0/ Supplementary Data S1
